# HIV-1 Escape from Small-Molecule Antagonism of Vif

**DOI:** 10.1128/mBio.00144-19

**Published:** 2019-02-26

**Authors:** Mark Sharkey, Natalia Sharova, Idrees Mohammed, Sarah E. Huff, Indrasena Reddy Kummetha, Gatikrushna Singh, Tariq M. Rana, Mario Stevenson

**Affiliations:** a Department of Medicine, University of Miami Miller School of Medicine, Miami, Florida, USA; b Department of Pediatrics, University of California San Diego School of Medicine, La Jolla, California, USA; University of Pittsburgh School of Medicine

**Keywords:** HIV-1, Vif, antiretroviral agents, antiretroviral resistance, APOBEC

## Abstract

The HIV-1 accessory protein Vif, which counteracts the antiviral action of the DNA-editing cytidine deaminase APOBEC3G (A3G), is an attractive and yet unexploited therapeutic target. Vif reduces the virion incorporation of A3G by targeting the restriction factor for proteasomal degradation in the virus-producing cell. Compounds that inhibit Vif-mediated degradation of A3G in cells targeted by HIV-1 would represent a novel antiviral therapeutic. We previously described small molecules with activity consistent with Vif antagonism. In this study, we derived inhibitor escape HIV-1 variants to characterize the mechanism by which these novel agents inhibit virus replication. Here we show that resistance to these agents is dependent on an amino acid substitution in Vif (V142I) and on a point mutation that likely upregulates transcription by modifying the lymphocyte enhancing factor 1 (LEF-1) binding site. Molecular modeling demonstrated a docking site in the Vif-Elongin C complex that is disrupted by these inhibitors. This docking site is lost when Vif acquires the V142I mutation that leads to inhibitor resistance. Competitive fitness experiments indicated that the V142I Vif and LEF-1 binding site mutations created a virus that is better adapted to growing in the presence of A3G than the wild-type virus.

## Abstract (precis)

Although antiretroviral therapy can suppress HIV-1 replication effectively, virus reservoirs persist in infected individuals and virus replication rapidly rebounds if therapy is interrupted. Currently, there is a need for therapeutic approaches that eliminate, reduce, or control persistent viral reservoirs if a cure is to be realized. This work focuses on the preclinical development of novel, small-molecule inhibitors of the HIV-1 Vif protein. Vif inhibitors represent a new class of antiretroviral drugs that may expand treatment options to more effectively suppress virus replication or to drive HIV-1 reservoirs to a nonfunctional state by harnessing the activity of the DNA-editing cytidine deaminase A3G, a potent, intrinsic restriction factor expressed in macrophage and CD4^+^ T cells. In this study, we derived inhibitor escape variants to characterize the mechanism by which these novel agents inhibit virus replication and to provide evidence for target validation.

## INTRODUCTION

The Vif protein of human immunodeficiency virus type 1 (HIV-1) is one of several primate lentiviral accessory proteins that function as virulence factors to promote viral replication and persistence (1, 2). It is encoded by one of six HIV-1 accessory genes, including the Tat, Rev, Vpr, Vpu, and Nef genes, that are present in addition to the Gag, polymerase (Pol), and Env genes common to all nondefective retroviruses. Early studies demonstrated that Vif was essential for virus replication in primary lymphoid and myeloid cells but was dispensable for replication in a subset of immortalized T-cell lines (3–5). Cells have been classified as either permissive or nonpermissive based on susceptibility to productive infection by Vif-deleted HIV-1. Experiments using heterokaryons formed by fusion of permissive and nonpermissive cells suggested that nonpermissive cells express a dominant inhibitor of HIV-1 replication that is absent in permissive cells (6, 7). Shortly thereafter, CEM15 was isolated from nonpermissive CEM cells, shown to be sufficient to confer the nonpermissive phenotype, and identified as APOBEC3G (A3G), a single-stranded DNA cytidine deaminase (8). HIV-1 has acquired Vif to counteract the highly potent, intrinsic antiviral activities of APOBEC3 proteins, and A3G in particular, that are expressed in the natural cellular targets of HIV-1 infection (reviewed in reference 9). In the absence of Vif, A3G is packaged into virions and deaminates cytidines in the viral minus-strand DNA formed during reverse transcription, resulting in lethal G-to-A hypermutation (10–12). There is also evidence that A3G packaged into newly formed virions inhibits reverse transcription through a deamination-independent mechanism (13, 14). To counteract APOBEC3 inhibition, Vif recruits an E3 ubiquitin ligase complex to APOBEC3 proteins to promote their polyubiquitination and subsequent proteasomal degradation (15, 16). Because of the absolute dependence on Vif for viral replication in the host, Vif remains an attractive and yet elusive antiviral target. Small molecules that uncouple the binding of Vif to APOBEC, or that disrupt the formation of the E3 ubiquitin ligase complex, have the potential to act as novel antivirals by promoting the stability of APOBECs. Thus, agents targeting the biological activity of Vif would activate a potent natural defense that could expand clinical treatment options and possibly lead to better strategies aimed at eradication of viral reservoirs.

We previously described a small-molecule antagonist of Vif (RN18) that was identified in a cell-based assay aimed at identifying compounds that stabilize A3G in the presence of HIV-1 Vif (17). Initial screening was based on cotransfection of 293T cells with a yellow fluorescent protein fused to A3G and wild-type (wt) or Vif-deleted vectors. Using this system, small molecules with inhibitory activity were identified through increased yellow fluorescent protein (YFP) signal and nonspecific effects could be excluded based on comparison to the fluorescent signal in matched cotransfections using Vif-deficient vector. RN18 exhibited specific, A3G-dependent antiviral activity that was manifest only in nonpermissive cells (expressing A3G) and not in permissive cells devoid of A3G expression (17). On the basis of the structural scaffold of RN18, additional compounds have been synthesized and screened to identify analogs of RN18 with improved antiviral activity. These compounds have been used to generate inhibitor escape mutants that provide evidence for target validation and delineate mechanistic aspects of Vif antagonist resistance.

## RESULTS

**FIG 1:**
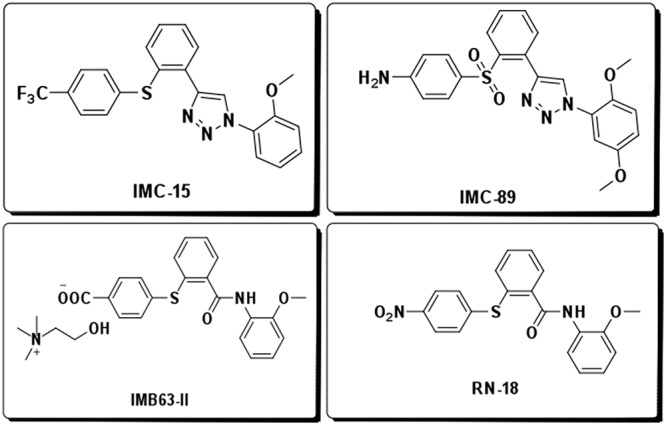
Structure of Vif antagonists. Inhibitor potency and metabolic stability were improved by structural modification of the lead Vif antagonist, RN18.

**FIG 2:**
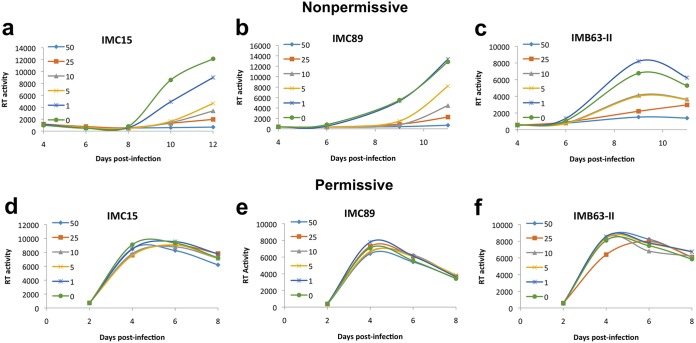
Analogs of RN18 inhibit replication in nonpermissive cells, but not in permissive cells. Nonpermissive H9 (a to c) or permissive MT4 (d to f) T-cell lines were treated overnight with various micromolar concentrations of each analogue before infection with equivalent amounts of HIV-1LAI. Cells were maintained 8 to 12 days in the presence or absence of inhibitors, and viral replication was monitored every other day by reverse transcriptase activity in culture supernatants. Data are representative of results from at least three independent experiments.

To enhance the potency and metabolic stability of RN18, we investigated the isosteric replacement of the amide functionality in RN18. We designed a series of conformationally restricted, biocompatible, and metabolically stable isosteric heterocyclic systems. We synthesized four test molecules by substituting the amide functionality in the lead molecule with isosteric heterocyclic systems such as 1,3,4-oxadiazole, 1,2,4-oxadiazole, 1,4-disubstituted-1,2,3-triazole, and 1,5-disubstituted-1,2,3-triazole. Synthesis of RN18 analogs containing isosteric heterocycles resulted in the discovery of a 1,2,3-triazole as a preferred scaffold for inhibition of HIV-1 Vif function and restoration of A3G levels in nonpermissive H9 cells (18). Further structure-activity relationship (SAR) studies were performed by synthesizing libraries of compounds with preferred scaffolds to identify a number of analogs with the desired antiviral activity profiles, i.e., antiviral activity in nonpermissive (H9) but not in permissive (MT4) cells (18). Three compounds, IMC15, IMC89, and IMB63-II, were prioritized because of their antiviral specificity and potency as assessed by spreading infections in a cell-based assay. Chemical modifications to RN18 leading to increased potency are presented in Fig. 1. HIV-1 replication was potently inhibited in a dose-dependent manner in nonpermissive cells (Fig. 2a to c), but no inhibition was observed in permissive cells (Fig. 2d to f). Half-maximal inhibitor concentrations (IC_50_s) were determined for the compounds and relative to those of RN18 (5.7 μM), each of the analogs exhibited increased potency (IMC15, 2.61 μM; IMB63-II, 2.15 μM; IMC89, 0.62 μM).

**FIG 3:**
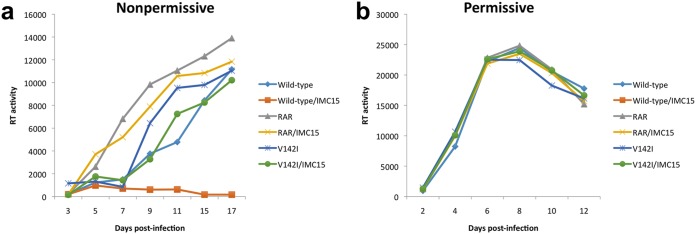
Replication profiles of wild-type, V142I Vif, or cloned RN18 analog-resistant (RAR) viruses in the absence or presence of IMC15 antagonist. Cells were infected with equivalent amounts of HIV-1LAI, and virus replication was monitored over time based on reverse transcriptase activity in culture supernatants. (a) Wild-type virus was sensitive to IMC15, while both the V142I Vif point mutant and RAR virus replicated efficiently in the presence of antagonist. (b) All viruses replicated similarly in permissive MT4 cells that did not express A3G. Data are representative of results from at least three independent experiments.

To validate the mechanism of action, each of the Vif antagonists were used to generate inhibitor escape mutants. Wild-type HIV-1LAI (GenBank accession number K02013) was passaged in nonpermissive H9 cells in stepwise increasing concentrations of the analogs to generate variants with the capacity to replicate in high concentrations of analogs. Previous experiments using inhibitor concentrations ranging from 1 to 50 μM showed dose-dependent reductions in wild-type virus replication and substantial inhibition at the lower concentrations used (Fig. 2). On the basis of these data, passage 1 H9 cells infected with wild-type HIV-1 were maintained in culture medium containing inhibitors at a 1 μM final concentration. Increases in inhibitor concentrations were gradual and dependent on the ability of the virus to overcome inhibition at a given concentration. Similar cultures were maintained in parallel without addition of the inhibitor to assess changes in the virus occurring spontaneously during prolonged H9 cell passage. Transition from one passage to the next was initiated when the level of virus production from treated cells was like that observed for cultures not maintained under conditions of selective pressure. The durations of the passages differed depending on the inhibitor concentration used but generally fell within a range of 2 to 4 weeks. New passages were initiated by exposing uninfected H9 cells to filtered culture supernatants from the previous passage with the addition of elevated concentrations of drugs. After 16 passages (**∼**11 months), viruses had the capacity to replicate efficiently in H9 cell cultures containing 100 μM IMC15, IMC89, or IMB63-II. Replication profiles were similar for all analog-resistant viruses, and representative data utilizing IMC15 are presented for nonpermissive (Fig. 3a) and permissive (Fig. 3b) cell infections. While virus passaged in the presence of analogs exhibited resistance to those analogs, the analog-resistant viruses remained sensitive to RN18. The IC_50_ values for wild-type and V142I (IMC15-resistant) viruses were 2.6 μM and >25 μM, respectively. In contrast, The V142I mutation did not affect the IC_50_ for RN18, which was ∼5.5 μM for both the wild type and the V142I variants. For comparison, the IC_50_ values for the nonnucleoside reverse transcriptase (RT) inhibitor (NNRTI) efavirenz was <1 nM for the wild-type, RN18 analog-resistant (RAR), and V142I variants.

**FIG 4:**
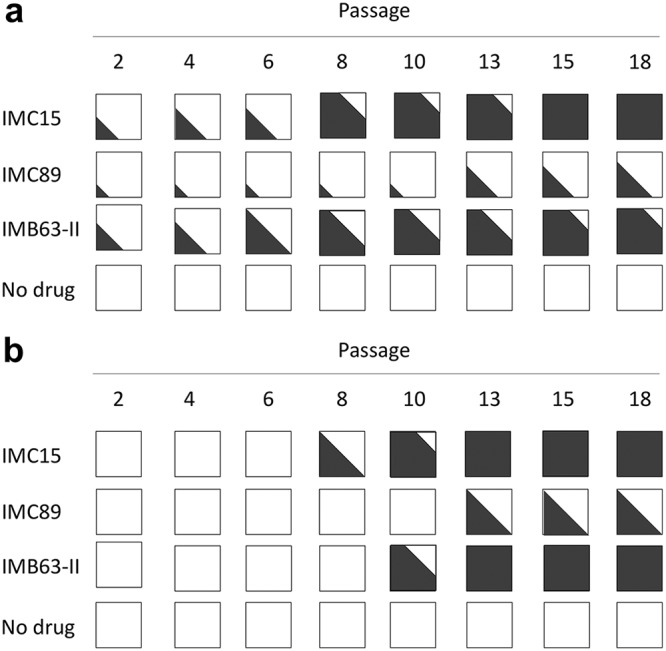
Vif inhibitor escape was achieved through mutations in Vif (V142I) and the LEF-1 binding site (C9007A nucleotide transversion). Viral sequences of Vif (a) and the LTR (b) were analyzed at each passage, and relative amounts of wild-type (white) and variant sequences (black) were estimated based on trace file peak heights.

Since we expected resistance to a Vif antagonist to develop because of changes in Vif, the *vif* gene was sequenced throughout the culture period. At alternating passages, the *vif* gene was amplified from cell cultures containing inhibitors and sequenced to detect changes that might confer resistance. Purified viral RNA was amplified by RT-PCR using primers that flank Vif, and amplification products were purified and submitted for Sanger sequencing by Genewiz (South Plainfield, NJ). Sequence trace file peak heights were used to estimate the relative amounts of wild-type and mutant virus present in the cultures at the alternating passages. For example, representative results showed that wild-type V142 (codon GTA) transitioned to V142I (codon ATA) in the presence of increasing amounts of IMC15 during long-term passage (see Fig. S1 in the supplemental material). As summarized in Fig. 4a, exposure to each of the RN18 analogs resulted in the rapid selection of isoleucine for valine at position 142 in Vif. Interestingly, this valine is part of a hydrophobic interaction domain immediately adjacent to the Vif SOCS box that promotes binding to EloC (15, 16). There is a binding pocket for V142 in EloC, and alteration at that site might influence the binding of Vif to EloC. To further explore the binding of Vif to EloC, structural modeling was used to define antagonist binding with and without the altered side chain at Vif amino acid 142.

10.1128/mBio.00144-19.1 FIG S1 Trace file peak heights determine the relative amounts of wild-type and V142I mutant viruses present in longitudinal samples of H9 culture supernatants in the absence or presence of Vif antagonist. The wild-type V142 (GTA) codon was maintained throughout the culture period in the absence of inhibitor, while V142I (ATA) emerged over time in the presence of increasing amounts of IMC15 Vif antagonist. Blue arrows indicate the first nucleotide of the Vif V142 codon that mutates due to inhibitor selective pressure. Download FIG S1, TIF file, 2.1 MB. Copyright © 2019 Sharkey et al. 2019 Sharkey et al. This content is distributed under the terms of the Creative Commons Attribution 4.0 International license.

**FIG 5:**
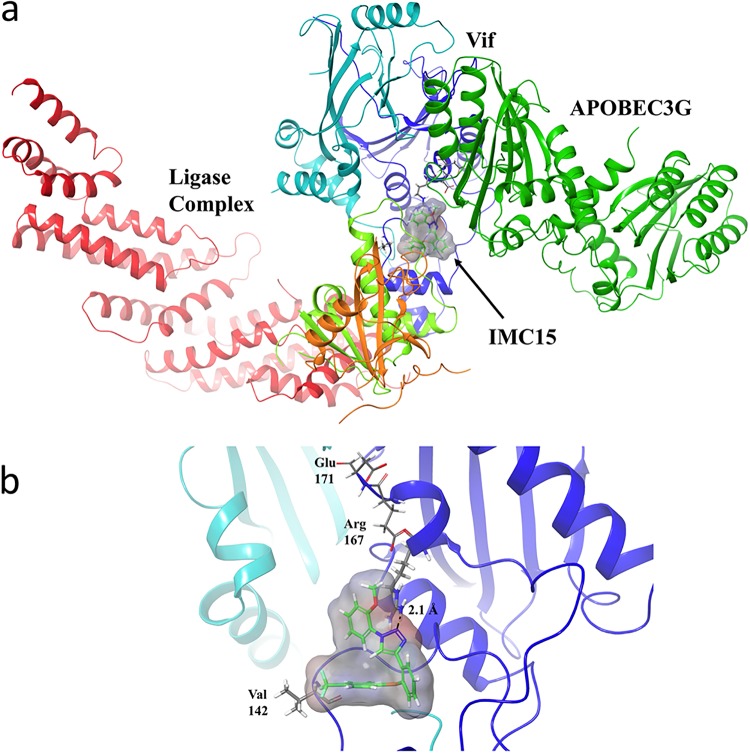
*In silico* model of IMC15 bound to Vif in complex with the E3 ligase and A3G. IMC15 is rendered in stick form, with surface density represented in gray (a). Vif is rendered as a dark blue ribbon, with EloB in orange, EloC in light green, CBF-β in light blue, Cullin 5 in red, and A3G in dark green. IMC15 binds Vif in proximity to the APOBEC binding region, destabilizing Vif-APOBEC interactions. IMC15 makes primary stabilizing Van der Waals interactions with V142 and π-cation interactions with Arg 167 (b). IMC15 is rendered in stick form, with surface density shown in gray. Vif is rendered as a dark blue ribbon, with CBF-β as a light blue ribbon.

To visualize the interactions of IMC15 with the Vif-CBF-β-CUL5-EloB-EloC complex, the compound was docked to a crystal structure of the complex (PDB identifier [ID] 4N9F) (19) using the molecular modeling program Schrödinger (20–22). The docking grid was defined as a 25-Å cube centered on coordinates x = 25.19, y = −14.1, and z = −96.93, near V142 of Vif. As shown in Fig. 5, IMC15 is predicted to bind to the complex primarily through interactions with Vif, although some Van der Waals interactions with loop 5 of EloC are also observed. IMC15 is primarily stabilized through π-cation interactions with Arg 167 of Vif. To understand how IMC15 might disrupt binding of Vif to A3G, the molecular modeling program Coot (23) was used to model the Vif-APOBEC interface, aligning residues from each protein known to be critical for binding (PDB ID 4N9F and 5K81) (19, 24, 25). Fig. 5a shows the modeled Vif-CBF-β-CUL5-EloB-EloC complex with A3G, where IMC15 clearly interrupts binding interactions between Vif and A3G. Further detailed analyses revealed that primary Van der Waals interactions were stabilized with Val 142 and π-cation interactions with Arg 167 (Fig. 5b).

To understand how the V142I mutation would affect binding of IMC15 to Vif, the mutation was generated using the Prime module of Schrodinger and IMC15 was again docked to the protein using a 25-cubic-Å grid centered on the coordinates x = 25.19, y = −14.1, and z = −96.93. In this instance, no favorable binding interactions were observed, suggesting that IMC15 is unable to bind to the site with that mutation. Loss of inhibitor binding would restore the ability of Vif to mediate the degradation of A3G, contributing to the observed resistance to the Vif antagonists.

Similarly, RN18 was docked against the Vif-CBF-β-CUL5-ELOB-ELOC complex to compare its predicted binding pose with that of IMC15. As shown in Fig. S2, the predicted binding pose for RN18 showed only weak Van der Waals interactions with Vif and was stabilized through interactions with EloC, namely, electrostatic interactions with Glu 92 and a weak hydrogen bond with the backbone of Ile 95. Modeling with the V142I Vif mutant complex also indicated little change in the expected binding mode of RN18. The Van der Waals contacts observed between RN18 and EloC in the wt Vif complex are maintained with the V142I mutant complex, with only a slight change in orientation of the anisole ring in RN18 observed. No change in contacts was expected between V142 and the V142I mutation. Therefore, it is likely that RN18 binds to a site that is different from that mediating IMC-15 binding on the Vif-CBF-β-CUL5-ELOB-ELOC complex.

10.1128/mBio.00144-19.2 FIG S2 RN18, the parental Vif antagonist, does not bind Vif with high affinity. RN18 is rendered in stick form, with surface density represented in gray. Vif is rendered as a dark blue ribbon, with CBF-β in light blue. Strong interactions were not observed upon modeling RN18 at the IMC15 binding site for either wild-type Vif (A) or V142I mutant Vif (B). Download FIG S2, TIF file, 2.3 MB. Copyright © 2019 Sharkey et al. 2019 Sharkey et al. This content is distributed under the terms of the Creative Commons Attribution 4.0 International license.

**FIG 6:**
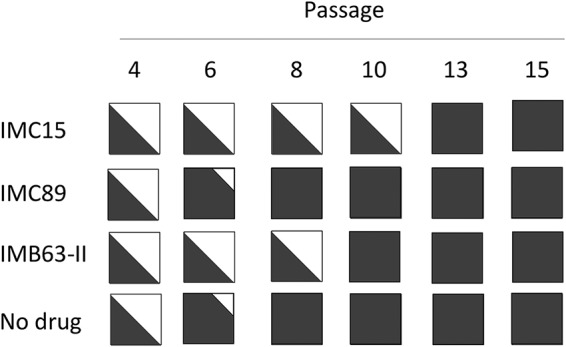
Vpr was rapidly inactivated in all H9 cell cultures due to a premature stop codon. Viral sequences of Vpr were analyzed at different passages, and relative amounts of wild-type sequences (white) and mutated sequences (black) were estimated based on trace file peak heights.

To identify additional changes that might contribute to inhibitor escape, subgenomic regions of viral RNA from several passages were amplified and sequenced. Sequencing revealed polymorphisms that were not present in the control virus that could potentially contribute to the resistance phenotype. A single nucleotide change in the long terminal repeat (LTR) converted the last cysteine residue in Nef to a stop codon. In addition to changing Nef, the C-to-A transversion at genomic position 9007_LAI_ potentially modifies an enhancer element important for transcriptional activation (26, 27). Treatment with each of the inhibitors resulted in the selection for this mutation, although with delayed kinetics relative to the V142I substitution in Vif (Fig. 4b). The polymorphism maps to the binding site for lymphocyte enhancing factor-1 (LEF-1; Los Alamos HIV sequence database) and may function to increase the transcriptional activity of the virus (28). Vpr was also inactivated during passage of virus in our H9 cell cultures containing each of the analogs. However, it is unclear whether this contributes to the resistance phenotype since Vpr was also lost during passage of virus in the absence of antagonist (Fig. 6). Vpr is cytostatic, and inactivation of *vpr* during long-term culture of HIV-1 is a well-recognized phenomenon (29).

Additional mutations were noted at some passages but appeared to be stochastic. The V142I Vif and C9007A and Vpr null changes emerged early and were maintained throughout the culture period (Fig. 4 and 6). Since all three antagonists drove the development of the same mutations, a single full-length virus clone was generated and sequenced. Virus collected at passage 22 in H9 cell culture containing 100 μM IMB63-II was used to infect fresh H9 cells. Total DNA was purified from cells 10 days postinfection, and two halves of the proviral DNA were PCR amplified and recombined to generate a full-length virus clone. In addition to the key mutations, several other changes were found within the cloned genome; a complete list is presented in Table 1.

**TABLE 1:** Complete set of all mutations within the cloned RN18 analog-resistant virus

HIV-1_LAI_ genomic region	Mutation(s)	Notes
LTR	C17T, C9007A	TAR^*a*^ element stem (C17T), LEF-1 binding site (C9007A)
Gag	E12K, E40K, I223T, R361K	Protease inhibitor resistance
Pol p66	L491V	RNaseH
Vif	V142I	Elongin C, A3G binding
Vpr	W38stop	Vpr inactivation
Env	N306K, E434K, I520M, R538A, Q555H, A583T, K660E	Likely inherent Env variability
Nef	E59K, C206stop	Loss of last Nef amino acid

a TAR, *trans*-activation response element.

**FIG 7:**
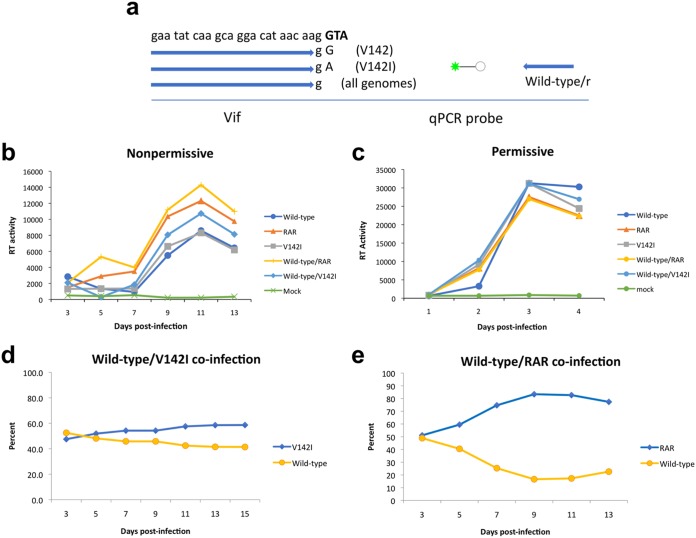
Viruses harboring resistance mutations exhibit a fitness advantage over wild-type virus in cells expressing A3G. (a) Selective primers were designed to generate allele-specific qPCR assays to measure relative amounts of viruses. (b and c) The codon for valine 142 is shown in bold. Replication profiles were generated in nonpermissive (b) and permissive (c) cells infected with single virus stocks or equivalent amounts of wild-type and mutant virus stocks. (d and e) The relative percentages of viruses in dual infections of nonpermissive H9 cells (d and e) with valine 142 (V142) (d) and RAR (e) were determined by allele-specific quantitative PCR of cDNA generated from viral RNA isolated from culture supernatants over time. Data are representative of results from at least three independent experiments.

We noted that the replication kinetics of the cloned analog-resistant virus appeared to be accelerated relative to that of wild-type virus (Fig. 3a). This difference is likely to have been A3G dependent, since the replication kinetics of wild-type and analog-resistant viruses were identical in permissive cells (Fig. 3b). This was surprising since wild-type virus should completely nullify A3G and, as such, should have the highest degree of fitness in nonpermissive cells. To investigate further, we measured the relative fitness levels of the parental and analog-resistant variants in pairwise competition experiments in nonpermissive H9 and permissive MT4 cells. In addition to the antagonist-resistant clone, a Vif V142I point mutant virus was included in the analysis. Cells were also infected with virus stocks singly to demonstrate replicative competence. As in the previous experiments, all viruses replicated similarly in permissive MT4 cells (Fig. 7c), while virus production levels varied in nonpermissive H9 cells such that analog-resistant virus cloned from late-passage culture exhibited enhanced replication kinetics relative to the wild-type virus (Fig. 7b). For dual infections, virions from culture supernatants of H9 cells were collected at 2-day intervals and the relative amounts of wild-type and antagonist-resistant viruses were measured by specific, quantitative PCR (qPCR) of cDNAs using primers that discriminated between the different alleles (Fig. 7a). The Vif V142I point mutant was modestly more fit than the wild-type virus in H9 cells (Fig. 7d), but the analog-resistant virus rapidly predominated in H9 cell coinfections (Fig. 7e).

## DISCUSSION

In this study, we identified and evaluated inhibitors of HIV-1 Vif function *in vitro* and delineated the mechanism of action of these novel antiretroviral compounds involving the binding of Vif to EloC. To our knowledge, this is the first report describing HIV-1 escape from small-molecule inhibition of Vif activity. Each of the Vif antagonists used in this study drove the rapid selection for the same polymorphism within the Vif SOCS box, while the no-treatment control virus was unchanged for the culture duration. On the basis of crystallographic data, one would predict that changes at Vif V142 could impact binding to EloC (15). Vif targets A3G for polyubiquitination by mimicking cellular SOCS box-containing proteins via its BC box that binds to EloB and EloC. Residues in the C-terminal region of Vif, including V142, make side chain interactions with helix 3 and helix 4 of EloC, and the protein surface of EloC shows a binding pocket for V142 (15). This is consistent with our structural modeling, which places IMC15 at this Vif-EloC binding interface, and the modeling suggests that inhibitor binding may be disrupted when valine is replaced by isoleucine at position 142. In further support of this, a small molecule (VEC5) identified by virtual screening and validated by subsequent biochemical analysis binds at the same site and exhibits antiviral activity by disrupting the Vif-EloC interaction (30, 31).

The complete genome of the cloned inhibitor-resistant virus was sequenced, and several mutations, in addition to the Vif V142I, were identified. Considering the location of the mutations in the context of previously published work, the Vpr null mutation and the C9007A transversion may contribute to high-level resistance to the Vif antagonists used here.

Hache et al. reported that Vif-deleted HIV-1 evolved to replicate in nonpermissive cells due to Vpr inactivation and a single mutation in the LTR promoter that increased transcription levels (32). It was postulated that Vpr inactivation was required to eliminate a Vpr-interacting factor that facilitates restriction by A3G, since Vpr knockout was not observed in control cells not expressing A3G (32). In contrast, Vpr was mutated in our system in each of the cultures containing Vif antagonists as well as in the no-treatment culture. In general, T cells infected *in vitro* with an HIV-1 isolate that contains an intact Vpr gene fail to divide due to the cell cycle arrest properties of Vpr and eventually die (29, 33, 34). Although establishing cell lines chronically infected with HIV-1 is possible, most produce virus particles with mutations in Vpr that render the protein nonfunctional (29, 35). Interestingly, the same Vpr nonsense mutation that emerged in our cultures had previously been detected at high frequency in HIV-1 serially passaged *in vitro* and in peripheral blood mononuclear cells (PBMC) from chronically infected individuals and may represent a “hot spot” for Vpr inactivation (35). Further study will be required to elucidate whether inactivation of Vpr in our system contributes to the observed inhibitor-resistant phenotype.

The C9007A transversion in the distal region of the LTR promoter suggests that the mutation could elevate proviral transcriptional activity. It maps to the binding site for LEF-1, a regulatory protein that shares homology with high-mobility group protein-1, which was previously shown to potently activate the T cell receptor α enhancer in T lymphocytes (36). C9007A also results in a one-amino-acid truncation of Nef, but an exhaustive search of the literature failed to find a description of an important biological property for the terminal cysteine in Nef or to indicate that its loss would have an impact on Nef function. The mutation emerged in each of the Vif antagonist cultures but not in virus replicating in the untreated control cells. On the basis of sequencing data, selection for the enhancer mutation was delayed relative to that of the Vif mutation and likely represents a secondary modification that may elevate virus production to further overcome the restriction by A3G, which was previously reported to be a mechanism by which Vif-deleted HIV-1 can evolve to replicate in nonpermissive cells expressing A3G (32). Results of efforts to directly compare the levels of transcriptional activity of wild-type and variant promoters using reporter plasmid constructs were uninformative. Measuring the relative levels of promoter activities may require a nucleosome-assembled *in vitro* approach, since LEF-1 transactivation is a chromatin-dependent process whereby LEF-1 functions to effectively counteract nucleosomal repression of HIV-1 transcription and similar effects are not observed using transfected naked plasmid DNA (28).

Although attempts to measure relative levels of promoter activities were unsuccessful, the cloned virus exhibiting the resistance phenotype clearly possessed a growth advantage in infected cells relative to the wild-type virus. This suggests that, in the context of wild-type virus, Vif does not completely subjugate A3G and that even wild-type HIV-1 is subject to the antiviral effects of A3G. This is supported by studies demonstrating the presence of proviruses with evidence of APOBEC-mediated editing in HIV-1-infected individuals (37, 38). An inability of wild-type HIV-1 to completely neutralize A3G might be advantageous to viral replication *in vivo* since it may contribute to sequence evolution and adaptation to environmental pressures such as antiviral immunity.

In summary, we have identified and evaluated new HIV-1 Vif antagonists on the basis of the structure of a lead compound (RN18) that possesses increased solubility and elevated potency. Mutations contributing to HIV-1 escape from these Vif antagonists localize to a motif that is essential for Vif function, demonstrating that the inhibitors specifically target the Vif-APOBEC axis. Structural modeling strongly supports these conclusions and suggests that the loss of inhibitor binding contributes to the development of resistance. Further optimization based on structure-function relationships, especially in the context of the Vif-EloC interaction, has the potential to identify novel Vif inhibitors with clinical efficacy.

## MATERIALS AND METHODS

### Reverse transcriptase assay for HIV-1 replication.

H9 and MT4 cells were maintained in RPMI 1640 medium (Invitrogen) containing 25 mM HEPES and l-glutamine with 10% fetal bovine serum (FBS), 100 units/ml penicillin, and 100 μg/ml streptomycin (Invitrogen) at 37°C in a humidified incubator (5% CO_2_). Cells were seeded at 2 × 10^5^ per well in a 24-well plate, treated overnight with antiviral agents, and infected with HIV-1 (X4-tropic HIV-1LAI) at 2 × 10^5^ cpm reverse transcriptase units per well. Viral replication was monitored every day or every 2 days by measuring reverse transcriptase activity in culture supernatants.

### Measurement of relative viral fitness levels.

Cells were maintained as described in the section on the reverse transcriptase assay procedure and were infected using 2 × 10^5^ cpm reverse transcriptase units per well either with single-virus stock or a combination of wild-type and variant viruses (V142I Vif mutant virus or cloned RN18 analog-resistant virus). Cells were pelleted and washed 4 h after infection to remove residual inoculum. Viral replication was monitored every day (MT4 cell infections) or every 2 days (H9 cell infections) by reverse transcriptase assay. At each time point, total RNA was purified from 100 μl culture supernatant. RNA was converted to cDNA using random hexamers, and the relative amounts of wild-type and variant viral genomes were quantitated by allele-specific qPCR using HiDi *Taq* polymerase (myPOLS, Constance, Germany), which efficiently discriminates between single-base mismatches at the 3′ end of primers during annealing and extension. Forward primers are listed in Fig. 7a and started at nucleotide 5022 of HIV-1LAI (GenBank accession number K02013). Sequence of the common reverse primer is LAI^5393^GAGTAACGCCTATTCTGCTATGTCGA and that of the fluorogenic reporter probe is LAI^5236^56-FAM (6-carboxyfluorescein)-ACATTTTCC/ZEN/TAGGATTTGGCTCCATGGCTTAG3IABkFQ (Integrated DNA Technologies).

### Docking with Schrödinger.

*In silico* docking of IMC15 to the Vif-CBF-β-CUL5-EloB-EloC complex with APOBEC3G was performed using the Glide docking module of the Schrödinger 11.5 modeling software suite. The Vif-CBF-β-CUL5-EloB-EloC complex and APOBEC3G structures were first refined using Prime. Missing side chains and hydrogen atoms were resolved before docking, and the Optimized Potentials for Liquid Simulations All-Atom (OPLS) force field and the Surface Generalized Born (SGB) continuum solution model was used to optimize and minimize the crystal structures. Ligprep was used to generate a minimized three-dimensional (3D) structure for IMC15 using the OPLS 2001 force field. Docking was performed with Glide XP. The docking grid was defined as a 25-Å cube centered on the coordinates x = 25.19, y = −14.1, and z = −96.93), near V142 of Vif.

### Sequencing.

For each passage presented in Fig. 4 and 6, viral RNA was purified from 200 μl of culture supernatant and an aliquot was converted to cDNA by random hexamer priming. High-fidelity Phusion II polymerase (Thermo Fisher Scientific) was used for PCR amplification of the cDNA with primers specific for the LTR, Vif, and Vpr. Products were purified with silica columns (Qiagen), quantified, and used as templates for Sanger DNA sequencing (Genewiz). Sequencing data were analyzed using MacVector software, and sequence variants were identified by ClustalW alignments and visual evaluation of sequence trace files. For the full-length RN18 analog-resistant (RAR) viral clone, multiple overlapping sequence files were generated and assembled into a single contiguous file using Sequencher (Gene Codes Corporation).

### Data accessibility.

Alignments performed with the parental virus sequence (GenBank accession number K02013) guided annotation of the inhibitor-resistant virus sequence prior to submission to GenBank (accession number MH843935).
