# Supplementary figures and images for: HIV-1 Escape from Small-Molecule Antagonism of Vif
Source: mBio. 2019 Feb 26;10(1):e00144-19. doi: 10.1128/mBio.00144-19 (PMC6391917; doi:10.1128/mBio.00144-19)

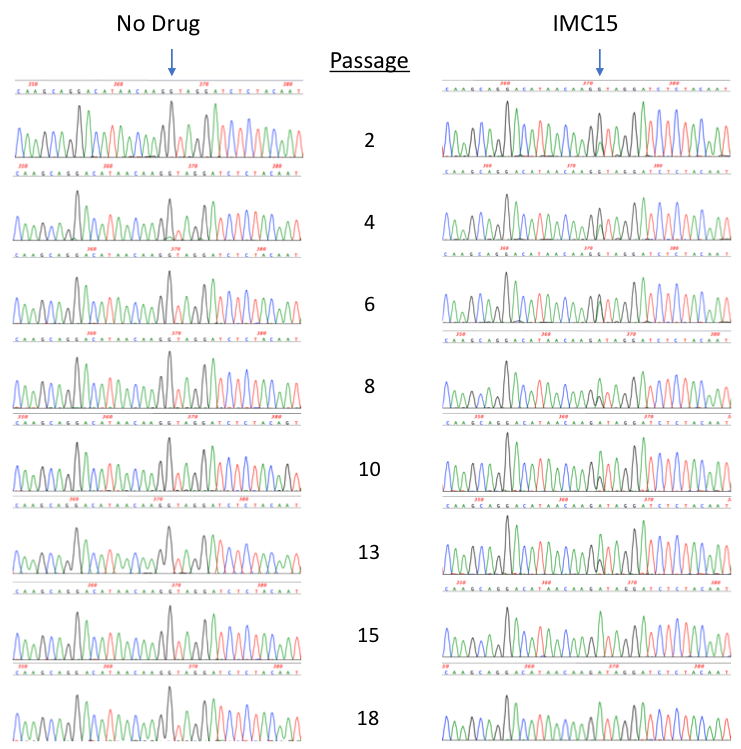

Supplement: FIG S1 [file mBio.00144-19-sf001.tif]

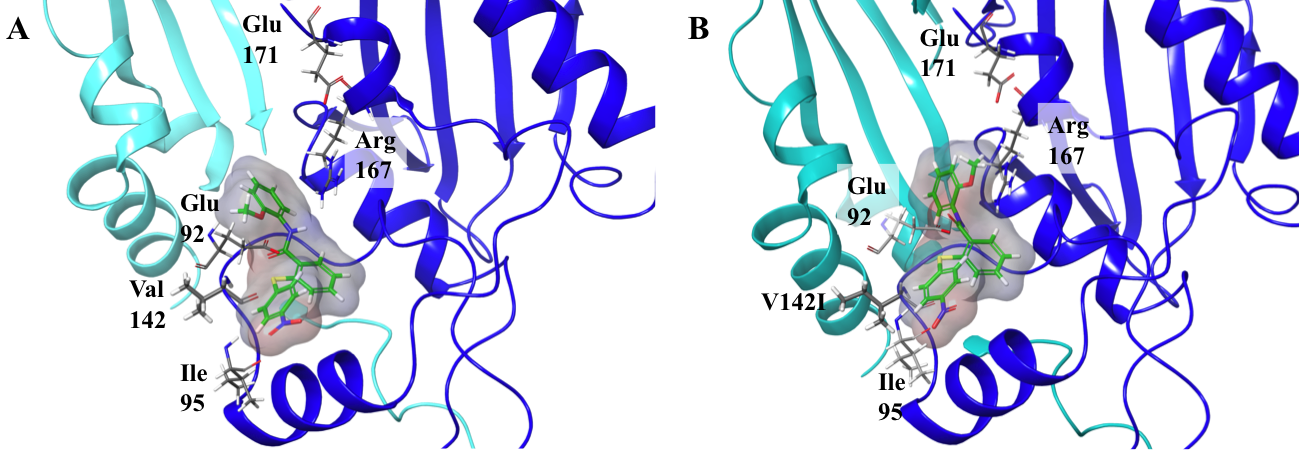

Supplement: FIG S2 [file mBio.00144-19-sf002.tif]
